# Modification of Polyurethane Sponge Based on the Thiol–Ene Click Reaction and Its Application for Oil/Water Separation

**DOI:** 10.3390/polym11122072

**Published:** 2019-12-12

**Authors:** Liping Liang, Yanyan Dong, Yuqing Liu, Xu Meng

**Affiliations:** 1 College of Life Science, College of Textile and Garment, Shaoxing University, Shaoxing 312000, China; liangliping0702@163.com (L.L.); dongyanyanys@163.com (Y.D.); Liuyq0605@163.com (Y.L.); 2 Key Laboratory of Clean Dyeing and Finishing Technology of Zhejiang Province, Shaoxing University, Shaoxing 312000, China; 3 Zhejiang Sub-center of National Carbon Fiber Engineering Technology Research Center, Shaoxing 312000, China

**Keywords:** PU sponge, functional, pollution technology, oil/water separation

## Abstract

A polyurethane (PU) sponge with hydrophobic/oleophilic property was prepared based on the thiol–ene click reaction, which was a simple method with only one step. Photopolymerization was induced through UV light on the sponge surface in a homogeneous solution containing polyethylene glycol diacrylate, pentaerythritol (mercaptoacetic acid) ester, 2-hydroxy-4′-(2-hydroxyethoxy)-2-methylpropiophenone, and octadecyl methacrylate. The as-prepared sponge possessed excellent selective for oil from various types of oil/water mixtures. It also had a high absorption capacity for toluene, >21 times its self-weight, and it had about 20 times its self-weight for vegetable oil, even after five extrusion–adsorption cycles, presenting a good recyclability. It created a new method to prepare oil/water separation sponge.

## 1. Introduction

In recent years, oil and organic solvent spills have caused worldwide concern due to the ensuing environmental pollution [1,2,3,4]. Thus, separating and recovering oil from water is becoming more and more important. 

To date, several technologies for oil/water separation have been developed, including gravity separation, membrane separation, chemical decomposition, and so on [5,6,7]. However, complex preparation steps, poor selectivity, and expensive costs have limited their practical application. Physical absorbent material for oil has been developed because of its mild operation condition and low cost, such as carbon fibers [8], microporous material [9], carbon nanotube [10], etc. Among them, PU sponge is regarded as a type of oil absorber material because of its three-dimensional (3D) network structure, high surface area, high mechanical strength, low density, and low price [11,12,13]. Inspired by the phenomenon of the lotus leaf, many researchers introduced substances with different properties on the skeleton of sponge to change the surface properties [14,15,16,17].

Recently, surface modification of sponge to change the property from hydrophilic and lipophilic to hydrophobic and lipophilic has attracted considerable attention [18,19]. A series of studies for hydrophobic and lipophilic PU sponges used in the separation of oil/water have been investigated. For example, Wang et al. [12] fabricated a superhydrophobic and superoleophilic carbon nanotube/poly(dimethylsiloxane) coated sponge for the continuous absorption and expulsion of oils and organic solvents from water surfaces. Nguyen et al. [11] fabricated a graphene-based sponge with both superhydrophobic and superoleophilic properties by a dip coating method. In recent literatures, although lots of modified sponges exhibited good absorption capacity, they were limited to practical applications due to complicated procedure and high cost. Thus, it is still importance for modifying sponge by a facile and mild method, which should be further investigated.

**Figure 1:**
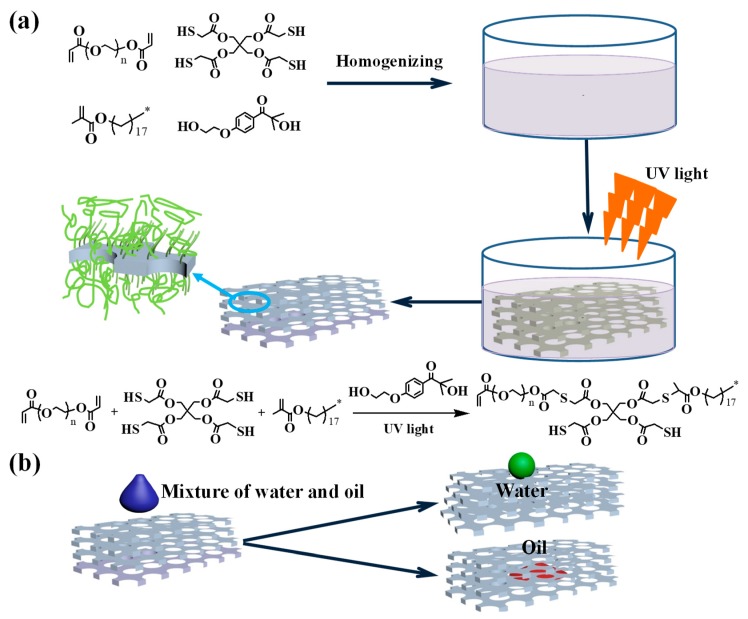
(**a**) Schematic illustration for the modification process of polyurethane sponge based on the thiol–ene click reaction, and (**b**) the application of oil/water separation.

Herein, a PU sponge with hydrophobic/oleophilic property was prepared based on the thiol–ene click reaction, which was a simple method with only one step. Photopolymerization was induced through UV light on the sponge surface in a homogeneous solution. Pentaerythritol (mercaptoacetic acid) ester and octadecyl methacrylate as the reactive monomer, polyethylene glycol diacrylate as a crosslinker, 2-hydroxy-4′-(2-hydroxyethoxy)-2-methylpropiophenone as a photoinitiator. The schematic illustration of the modification process for sponge based on the thiol–ene click reaction and the process of oil/water separation are presented in Figure 1. The as-prepared sponge possessed excellent selectivity for oil from various types of oil/water mixtures. It created a new method to prepare oil/water separation sponge.

## 2. Experimental

### 2.1. Materials

The sponge used was a commercially available polyurethane sponge. Polyethylene glycol diacrylate with a molecular weight of 600 g/mol (*M*n), analytical reagent grade pentaerythritol (mercaptoacetic acid) ester, octadecyl methacrylate, and 2-hydroxy-4′-(2-hydroxyethoxy)-2-methylpropiophenone were purchased from Aladdin Industrial Inc., Shanghai, China. Analytical reagent grade acetone, toluene, chloroform, hexane, and cyclohexane were obtained from Sinopharm Chemical Reagent Co., Ltd., Shanghai, China. Vegetable oil was obtained from COFCO, Shandong, China.

### 2.2. Modification of Polyurethane Sponge Based on the Thiol–Ene Click Reaction

Before the process of grafting polymerization, the sponge was cut into 15 mm × 15 mm × 3 mm pieces, and they were cleaned with ethanol and deionized water by ultrasonication for 30 min, respectively, which were dried completely using an oven at 60 °C, subsequently. Then, 200 μL of pentaerythritol (mercaptoacetic acid) ester, 0.3 g of polyethylene glycol diacrylate, 0.3 g of octadecyl methacrylate, and 0.05 g of 2-hydroxy-4′-(2-hydroxyethoxy)-2-methylpropiophenone were added to the beaker and dissolved evenly in acetone solution (30 mL). The blank sponge was soaked in the above solution, and then it was taken out and placed in a Petri dish after the adsorption was saturated. Subsequently, An UV lamp with 125 W was placed over the Petri dish kept a height of 10 cm for 2 h to initiate photopolymerization. Then, the sponge was flipped and continued to be exposed to light for 2 h. Finally, the sponge was taken out and dried at 65 °C for 10 min to obtain the modified sponge. The chemical reaction and the preparation process are shown in Figure 1.

### 2.3. Characterizations

The chemical structures of sponge before and after modifying were investigated by Fourier transform infrared spectroscopy (FTIR, 170SX, Nicolet, Madison, WI, USA). SEM images were obtained using a scanning electron microscope (SEM, EM-30, Coxem, Daejeon, Korea). Thermo analyses (TG) were investigated by TG/DTA 6300 equipment (TG, Nippon Seiko Co., Ltd., Tokyo, Japan). It was heated from 30 °C up to 600 °C with a heating rate of 20 °C/min under an air atmosphere. Contact angles were measured using an OCA50 machine (Data-Physics, Dataphysics, Stuttgart, Germany). The continuous oil/water separation process was investigated by a peristaltic pump (BT300-01, Longer Precision Pump Co., Ltd, Baoding, China). All photos were taken with a Canon camera. The absorption capacity was calculated based on our previously reported works [20].

## 3. Results and Discussion

**Figure 2:**
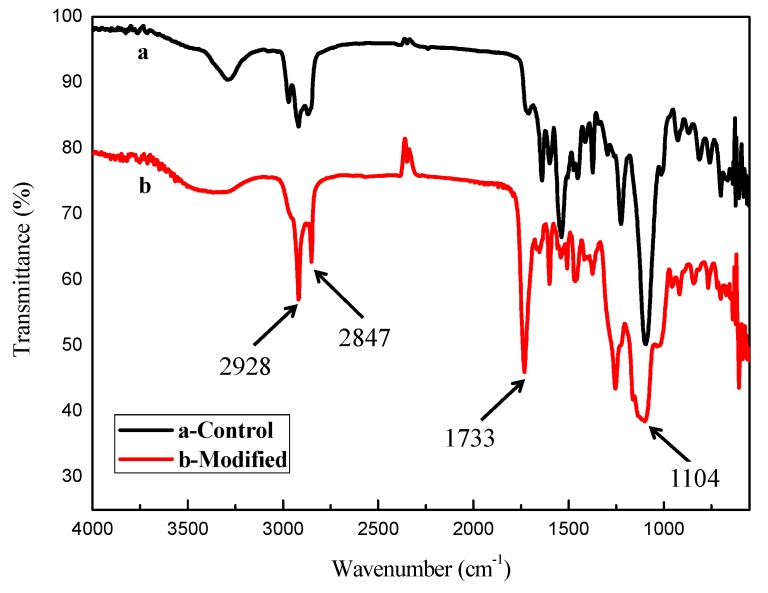
FTIR spectra of PU sponge: (**a**) control, (**b**) after modifying based on the thiol–ene click reaction.

The hydrophobic/oleophilic sponge was prepared based on the thiol–ene click reaction and the FTIR spectra of both pristine and polymer modified sponges are shown in Figure 2. The peaks appeared at about 2928 and 2847 cm^−1^ were attributed to the stretching vibrations of C−H presented in methyl and methylene groups. The peak at 1104 cm^−1^ was attributed to C–S–C groups, the S–H stretch locates at 2549 cm^−1^ didn’t appear, indicating that the functional groups were fully consumed during the reaction [21]. The vibration peak at about 1733 cm^−1^ was assigned to the C=O stretching vibrations. The results confirmed the graft reaction has been happened.

**Figure 3:**
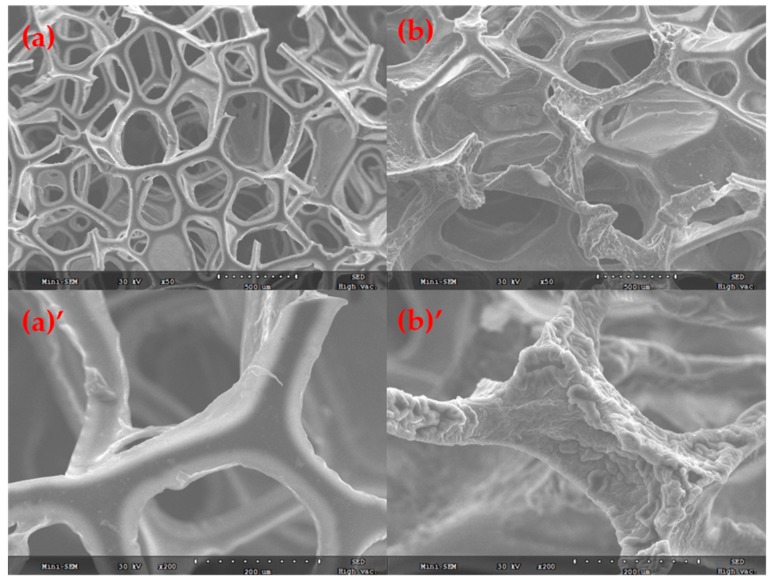
SEM images of PU sponge: (**a**) control, (**a′**) a microgram of blank sponge at high magnification resolution, (**b**) PU sponge after modifying, and (**b′**) a microgram of modifying sponge at high magnification resolution.

The morphologies of sponge before and after modification were studied by SEM. From Figure 3a,a′, it can be seen that the pristine sponge exhibits a 3D highly porous structure. The surface of the skeleton was smooth with an average pore size of about 500 μm. Meanwhile, the modified sponge displayed the same porous structure, as shown in Figure 3b,b′. However, compared to the blank sponge, the different is that uniformly coated polymers with evident rough surface covering the whole sponge, and this also proved that polymerization has occurred on the surface of the sponge. The rough surface is a prerequisite for the sponge’s hydrophobic/oleophilic property.

**Figure 4:**
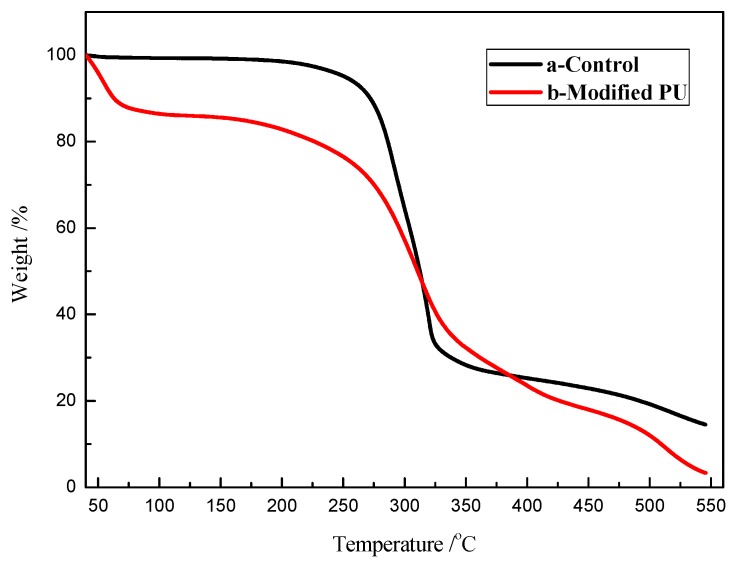
The TG curves of PU sponge before and after modifying: (**a**) control, (**b**) the modified sponge.

The thermal property of the coated polymers on the sponge surface is important because it determines the highest working temperature. The results of TG were shown in Figure 4, from the weight percentage curve, the modified sponge decomposed between 40 and 100 °C, which could be owing to the evaporation of water and degradation of cross-linked small molecules. Contrasted with a blank sponge (Figure 4a), the decomposition temperature of grafted sponge was reduced, and started decomposition at 140 °C with one big peak of weight loss (Figure 4b). It could be owing to decomposition of polymer grafted on sponge surface.

**Figure 5:**
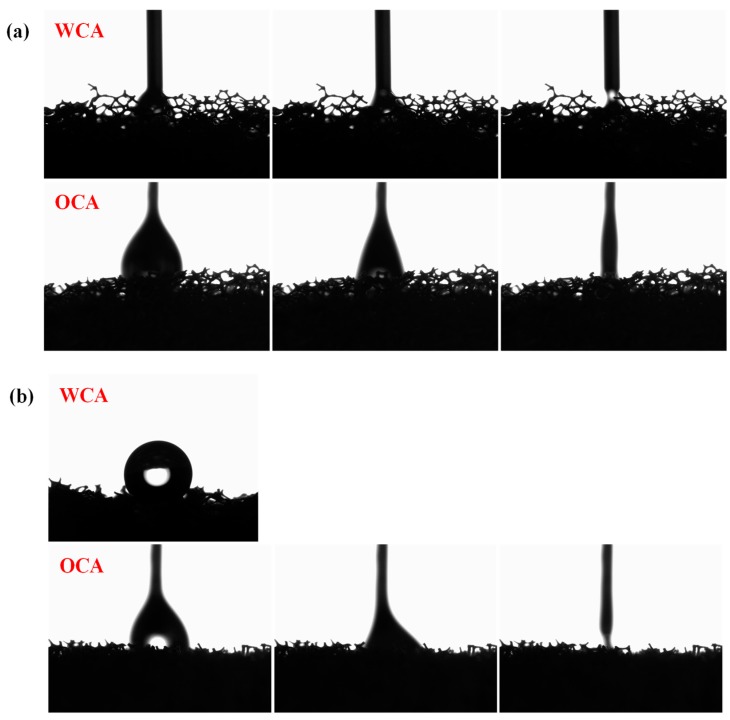
The water contact angle (WCA) and oil contact angle (OCA) of PU sponge before and after modifying: (**a**) the image of a water droplet and a chloroform droplet on the blank sponge, respectively; (**b**) the image of a water droplet and a chloroform droplet was on the modified sponge, respectively.

To evaluate the surface wettability of pristine and modified sponge, the contact angle was investigated, and the results were shown in Figure 5. The unmodified sponge could be easily wetted by both water and oil, it’s because of the superhydrophilicity and superoleophilicity of the pristine sponge, and the water and oil contact angles were both 0° (Figure 5a). However, the sponge changed its wettability after surface modification: the water droplet on the surface exhibited spherical shape with water contact angle of 159° (Figure 5b). By contrast, the value was higher than some reported works, such as the superhydrophobic sponge with a water contact angle of 157.0° ± 0.3° fabricated by Huang [22], and a thermo-responsive sponge with the highest water contact angle of 150° fabricated by Lei [23]. Meanwhile, the modified sponge could still be completely wetted by oil droplets with an oil contact angle of 0°. This indicated that the modified sponge had good hydrophobic/oleophilic properties.

**Figure 6:**
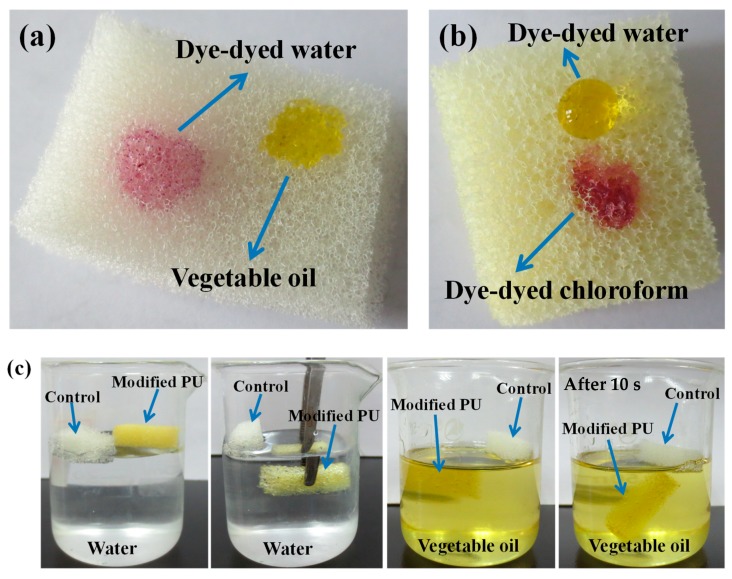
The wetting properties of the sponge before and after modifying: (**a**) images of a water droplet (dyed by dyestuff to be observed easily) and a vegetable oil droplet on the blank sponge; (**b**) images of a water droplet (dyed by dyestuff) and a chloroform droplet (dyed by dyestuff) on the modified sponge; (**c**) the states of the blank and modified sponge in water and vegetable oil.

To further compare their surface wettability properties, the appearance of water and oil droplets on the surface of sponge were investigated. Figure 6a shows the images of a water droplet (dyed by dyestuff to be observed easily) and a vegetable oil droplet on the pristine sponge. The unmodified sponge could be easily wetted by both water and oil because of the superhydrophilicity and superoleophilicity of the pristine sponge. However, based on the images of a water droplet (dyed by dyestuff) and a chloroform droplet (dyed by dyestuff) on the modified sponge in Figure 6b, the water droplet exhibited stable spherical shape on the surface of modified sponge because of the alkyl chain with the low surface tension. Meanwhile, the oil droplet could spread and permeate into its inner because of strong lipophilicity and the capillary force. When the sponge before and after modifying were put on water and oil surface (Figure 6c), it was found that the modified sponge could float on water surface during the entire test process because of its hydrophobicity and light weight. Furthermore, a mirror-like phenomenon was found when modified sponge was immersed in water using an external force, which was a result of a uniform air layer trapped between the hydrophobic surface and water. However, the modified sponge was put on the surface of vegetable oil, it could immerse into oil quickly after about 10 s because of its good oleophilic property.

**Figure 7:**
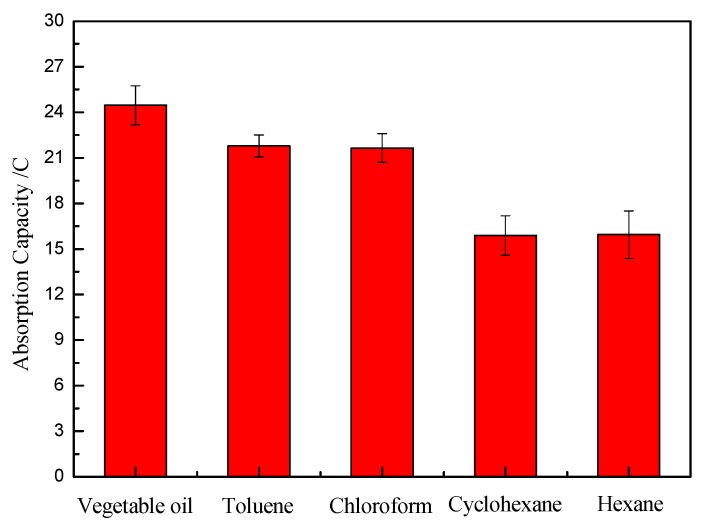
The absorption capacities of the modified sponge for various types of oils.

The absorption capacities of modified sponge for various types of oils and organic solvents were investigated, including vegetable oil, toluene, chloroform, cyclohexane, and hexane. The absorption capacity was calculated according to the following formula, *C* (%) = ((*n* − *n*_0_)/*n*_0_) × 100%, where *C* (%) is the absorption capacity, and *n*_0_ and *n* are the weight of the as-prepared sponge before and after absorption with oil, respectively. As shown in Figure 7, the results of all were above 15 times, and above 24 times for vegetable oil. The good absorption property was attributed to the strong capillary effect, high 3D hierarchical porous structure, and low surface energy. Thus, the as-prepared sponge exhibited greater potential in oil adsorption applications.

**Figure 8:**
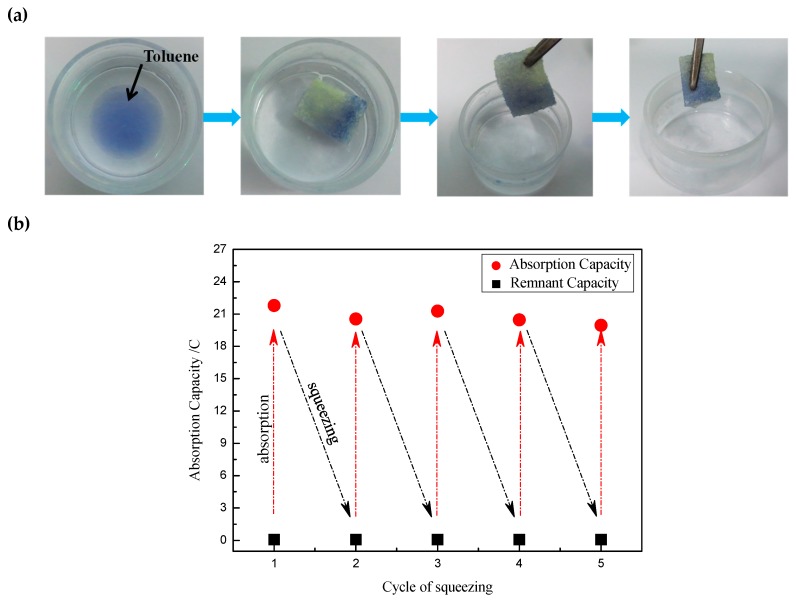
(**a**) The oil/water separation processes of the modified sponge and the collection of toluene (dyed by dyestuff) from water surface; (**b**) the changes of absorption capacity for vegetable oil after five squeezing cycles using the modified sponge.

The selective adsorption property for removal of oil from the oil/water mixture is important. Figure 8a presented the process of modified sponge collected toluene from water surface. When the as-prepared sponge contacted with toluene (dyed by dyestuff) on water surface, it could absorb toluene quickly upon repelling water. Furthermore, the oil-absorbed sponge floated on water surface because of its hydrophobic property. Then, toluene could be separated entirely only to remove the oil-absorbed sponge away and recycled by mechanical squeezing. It is very important that the sponge could be recycled in oil/water separation practical application. The changes of absorption capacity for vegetable oil after five squeezing cycles were shown in Figure 8b. During the test, we squeezed the saturated sponge with tweezers to squeeze the oil out of the sponge as completely as possible. The absorption capacity was calculated according to the following formula, *C* (%) = ((*n* − *n*_0_)/n_0_) × 100%. Where, *C* (%) is absorption capacity, *n*_0_ and *n* are the weight of as-prepared sponge before and after absorption with oil, respectively. The absorption capacity changed a little, and still could maintain about 20 times even if after five extrusion–adsorption cycles. These results demonstrated that the as-prepared sponge possessed a high absorption capacity and a good recyclability.

**Figure 9:**
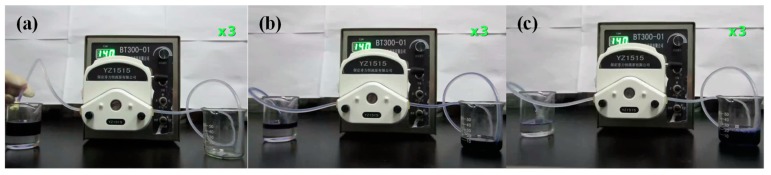
The process of continuous oil/water separation of modified sponge, which was assisted by a peristaltic pump. (**a**) Before separation; (**b**) during separation; (**c**) after separation.

The successive oil/water separation of as-prepared sponge was further studied using a peristaltic pump, as shown in Figure 9a. The device consisted of a peristaltic pump, and a rubber tube tied with modified sponge. Figure 9b displayed the separation process of toluene (colored for clear observation) from water. It showed that a stream of toluene was formed when turned on the peristaltic pump. Toluene could be separated continuously, and level of toluene was declined gradually (Video S1 in Supplementary Materials). After a few minutes, there was no remaining toluene to be found on the water’s surface. Furthermore, there was almost no water could be seen in the collected toluene (Figure 9c). The results indicated that as-prepared sponge showed high selectivity and good separation efficiency, and it would have the potential application in oil/water separation.

## 4. Conclusions

The hydrophobic/oleophilic sponge was successfully prepared based on the thiol–ene click reaction, which was a simple method with only one step. The as-prepared sponge possessed excellent selective for oil from various types of oil/water mixtures. It also had a high absorption capacity for toluene, >21 times its self-weight, and it had about 20 times its self-weight for vegetable oil even after five extrusion–adsorption cycles, presenting a good recyclability. It created a new method to prepare oil/water separation sponge. The as-prepared sponge will be a promising candidate material for the removal of oil pollutants from water because of the simple preparation process and superior properties.

## Supplementary Materials

The following are available online at https://www.mdpi.com/2073-4360/11/12/2072/s1, Video S1: video of successive oil/water separation process.
